# MTMEGPS: An R package for multi-trait and multi-environment genomic and phenomic selection using deep learning

**DOI:** 10.3389/fpls.2025.1674985

**Published:** 2026-01-05

**Authors:** Freddy Mora-Poblete, Javiera Valenzuela-Herrera, Matías Balach, Claudio J. Martínez-Araya, Carlos Maldonado

**Affiliations:** 1 Institute of Biological Sciences, University of Talca, Talca, Chile; 2 Centro de Genómica y Bioinformática, Facultad de Ciencias, Universidad Mayor, Huechuraba, Santiago, Chile

**Keywords:** deep learning, genomic selection, spectral information, molecular markers, multi-traits and multi-environments, hyperparameters optimization

## Abstract

Genomic and phenomic selection have transformed modern breeding by enabling data-driven prediction of complex traits. Deep learning (DL) can further enhance predictive ability by capturing nonlinear patterns that classical and Bayesian approaches often fail to represent. However, despite its potential, the adoption of DL in breeding programs remains limited due to its computational demands and the lack of accessible tools for users without extensive programming experience. This study introduces the *MTMEGPS* (Multi-Trait and Multi-Environment Genomic and Phenomic Selection), an R package that provides a streamlined end-to-end workflow for Uni- and Multi-Trait (UT and MT, respectively) and Uni- and Multi-Environment (UE and ME, respectively) genomic and phenomic prediction. The package supports data preparation, hyperparameter optimization, model training, and DL-based evaluation. To assess its performance, *MTMEGPS* was applied to the two default datasets included in the package: Maize (genomic data) and Eucalyptus (near-infrared spectroscopy, NIR, data), as well as to an independent publicly available multi-environment validation dataset. Across most scenarios, *MTMEGPS* showed superior predictive ability compared with all benchmark models, particularly under UT for the internal datasets and MT for the independent multi-environment dataset. Mean squared error (MSE) values were similar across models, all falling within a moderate range. Overall, these results demonstrate the efficiency and practical utility of *MTMEGPS* for genomic and phenomic selection, even in scenarios where prediction errors remain moderate.

## 1 Introduction

Classical plant breeding has evolved considerably over the last century, thanks to the combined action of the application of improved experimental designs, statistical methods and new phenotyping technologies (Sandhu et al., 2022). However, classical breeding has only demonstrated the potential to increase the annual rate of genetic gain of crops to approximately 1% for major cereals (Masuka et al., 2017; Cobb et al., 2019), which is insufficient to aim at securing a stable food supply for the growing human population (Montesinos-López et al., 2021a; Sandhu et al., 2022). This has pressured plant breeders to look for new ways to improve crop yields, involving a combination of innovative techniques such as next-generation sequencing, high-throughput phenotyping, and advanced statistical methods (Varshney et al., 2021).

Genomic selection (GS), proposed by Meuwissen et al. (2001), lies in leveraging genome-wide DNA variations alongside phenotypic information from an observed population to predict the phenotypic values of an unobserved population (Meuwissen et al., 2001; Montesinos-López et al., 2021a). This approach has been shown to explain a larger proportion of genetic variation and provides more accurate estimates of relationships among individuals, surpassing traditional pedigree-based methods, enabling breeders to achieve higher rates of genetic gain (Meuwissen et al., 2001; Montesinos-López et al., 2021b). A key advantage of GS is its ability to reduce breeding cycles while efficiently selecting complex quantitative traits. The practical application of GS in plant breeding began in 2007, when Bernardo and Yu (2007) demonstrated its superiority over marker-assisted recurrent selection in maize, which later spread to various crop species (Sandhu et al., 2022).

Despite the adoption of GS in plant breeding in the last two decades, the costs and efforts associated with genotyping thousands of selection candidates generated in each breeding cycle remain substantial (Zhu et al., 2021). Therefore, Rincent et al. (2018) proposed a novel approach termed phenomic selection (PS), which leverages high-throughput phenotyping data, such as near-infrared spectroscopy (NIR), as an alternative, non-destructive, and low-cost method to molecular markers for selection of superior individuals. In this sense, PS builds on replacing genomic markers with phenomic data, such as NIR spectra, to predict quantitative traits using statistical models. Notably, NIR-based PS has demonstrated prediction accuracies comparable to or even surpassing those achieved with molecular marker data in agricultural and forestry crops (Lane et al., 2020; Mora-Poblete et al., 2024; Maggiorelli et al., 2024; Roscher-Ehrig et al., 2024; Mieres-Castro et al., 2024).

The genomic selection methods have traditionally relied on univariate (Uni-Trait; UT) approaches, where individual traits are modeled independently. To address these limitations, multivariate (Multi-Trait; MT) selection approaches have been developed, which analyze multiple traits simultaneously and leverage genetic interrelationships to improve prediction accuracy (Tang et al., 2024). Furthermore, recent advancements have highlighted the potential of multi-environment (ME) approaches and their combination with MT (MTME), which integrate genotype-by-environment (GxE) interactions to increase prediction accuracy across diverse conditions (Montesinos-López et al., 2019; Sandhu et al., 2022).

Recent studies have shown that deep learning can outperform traditional GBLUP and Bayesian models in UT, MT, and MTME settings, particularly for nonlinear or low-heritability traits (Montesinos-López et al., 2018; Mora-Poblete et al., 2023; Mieres-Castro et al., 2024; Montesinos-López et al., 2025). DL models are nonparametric models capable of identifying complex data patterns and capturing non-linear relationships without relying on prior assumptions, which is particularly beneficial for low-heritability traits and complex interactions. However, challenges remain to be addressed, particularly in hyperparameter tuning (determining the number of layers, neurons, activation functions, and regularization rates), which is crucial for achieving robust predictions, and a program or package that facilitates the creation and training of DL models, particularly for individuals without a strong programming background (Rokis and Kirikova, 2022).

**Figure 1:**
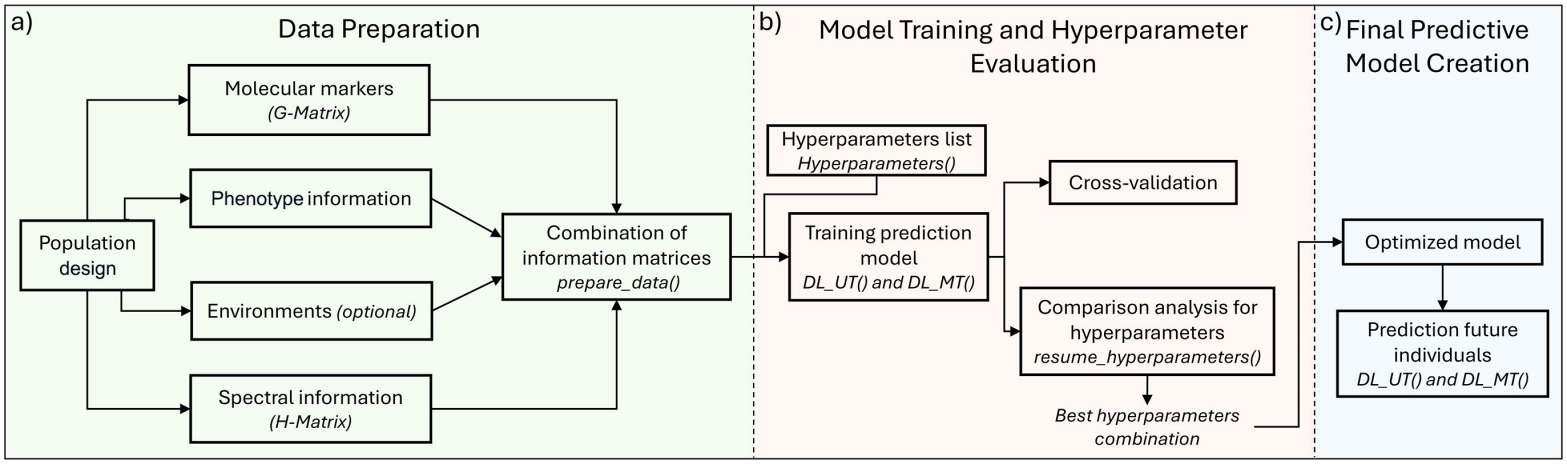
Workflow of the *MTMEGPS* package. **(a)** Data preparation: The *prepare_data()* function integrates phenotypic information with the genomic (G-matrix) or combined genomic–pedigree (H-matrix) relationship matrix, and with environmental data when applicable, generating the input dataset required for model training. **(b)** Model training and hyperparameter evaluation: The *DL_UT()* and *DL_MT()* functions train deep learning models while simultaneously evaluating multiple hyperparameter combinations through parallel processing. The *resume_hyperparameters()* function summarizes the performance of all tested configurations and identifies the optimal hyperparameter set. **(c)** Final predictive model: The *DL_UT()* and *DL_MT()* functions can then be used to generate predictions for new individuals based on the finalized model.

The methods for selecting superior individuals (GS and PS) combined with MTME approaches show great potential. However, issues related to the optimization required for large-scale implementation in breeding programs still need to be addressed. Despite recent advances, there is currently no open-source tool that allows for end-to-end DL-based genomic and phenomic selection, combining MT and ME data. This gap hinders adoption by breeding programs lacking strong programming resources. To address this limitation, this study introduces *MTMEGPS* (Multi-Trait and Multi-Environment Genomic and Phenomic Selection), an R package that allows building and training a DL model using UT, MT, UE, and ME approaches, as well as identifying the best combination of hyperparameters for the model. *MTMEGPS* is available on GitHub: https://github.com/DeepScienceLabCM/MTMEGPS-package. The package is compatible with Linux, MacOS and Windows, ensuring accessibility for a wide range of users. *MTMEGPS* is an open-source, fully documented, and computationally optimized tool designed to predict complex traits from spectral data (e.g., near-infrared spectroscopy, NIRs) or genomic data (e.g., single-nucleotide polymorphisms, SNPs). The package provides three main functions, each implemented with parallel processing: (i) preparation of the input database, which guides the user in structuring the data according to the selected approach (UT, MT, UE, or ME); (ii) hyperparameter optimization, which identifies and summarizes the best-performing hyperparameter combinations; and (iii) construction, training, and validation of the predictive model (**Figure 1** and Codes.R). This package represents a significant advance by integrating molecular and spectral data within a DL framework for the first time, incorporating MT and ME approaches with a DL model, thereby making it an indispensable tool for accelerating genetic progress and optimizing selection decisions in breeding programs. The package name *MTMEGPS* is an acronym for Multi-Trait and Multi-Environment Genomic and Phenomic Selection. For clarity, the suffix ‘GPS’ refers to ‘Genomic and Phenomic Selection’ and is not related to Global Positioning System or geolocation data, which are not incorporated into the package.

## 2 Materials and methods

### 2.1 Internal datasets for software validation and demonstration

A subset of the Maize database (molecular information) obtained from Mora-Poblete et al. (2023) and the Eucalyptus database (spectral information) from Mieres-Castro et al. (2024) were used to illustrate the application of *MTMEGPS* in the genomic (SNPs) and phenomic (NIRs) selection analysis. The Maize dataset comprises 258 tropical maize inbred lines from the core germplasm collection of the State University of Maringá, located in Paraná, Brazil. The phenotyping was constructed using phenotypic data collected across two sites within Paraná: Cambira and Sabaudia (season 2017-2018). The following flowering traits were evaluated: Female Flowering time (FF), Male Flowering time (MF), and Anthesis-Silking Interval (ASI) calculated as the difference between MF and FF (Maldonado et al., 2020). The SNPs molecular markers were discovered through genotyping by sequencing (Elshire et al., 2011). The Eucalyptus dataset consisted of 1208 individuals from La Poza, Purranque, in the administrative region of Los Lagos, Chile (forest seed orchards of Semillas Imperial SpA). Phenotypic traits of industrial interest related to wood production-related traits were assessed in 9-year-old trees: plant height (PH), diameter at breast height (DBH), and stem volume (VOL). The absolute reflectance of leaves was measured following the methodology of Castillo et al. (2008) using a NIR spectrometer (NIRQuest512 spectrometer, Ocean Optics, Inc., Orlando, FL, USA), and HL-2000-HP-FHSA light source, and a 3.18 mm diameter bifurcated optical fiber (QR600-7-VIS- 125F). The raw SNP and the G-matrix (relationship matrix estimated following VanRaden, 2008) were utilized to represent genomic information. Likewise, spectral data and the H-matrix (relationship matrix estimated as per Rincent et al., 2018) were employed to represent high-dimensional phenotypic information.

### 2.2 Independent multi-environment maize validation dataset

The datasets analyzed in this study were obtained from the Genomes to Fields (G2F) initiative (www.genomes2fields.org). The dataset comprises 135 unique maize hybrids evaluated across nine experimental sites during the 2018 growing season (https://doi.org/10.25739/anqq-sg86). Phenotypic measurements were collected following standardized protocols provided by the G2F consortium, as detailed in the accompanying documentation available on the project website.

The traits evaluated in this study included plant height (distance from the plant base to the ligule of the flag leaf), ear height (distance from the soil surface to the primary ear-bearing node), and grain yield (measured in bushels per acre at 15.5% grain moisture, assuming 56 lbs per bushel and using the net plot area excluding alleys). Data points falling outside biologically reasonable ranges were treated as missing. Individuals lacking data for any of the nine sites were excluded from subsequent analyses. The samples of the evaluated hybrids were genotyped using the Practical Haplotype Graph (PHG) framework (Bradbury et al., 2022). SNPs markers that were monomorphic or had a call rate below 90% were filtered out. Additionally, SNPs with a minor allele frequency (MAF) less than 0.05 were removed. After filtering, a total of 939,128 high-quality SNPs were retained for downstream analyses in the 135 maize hybrids.

### 2.3 Preparation of the database for UT, MT, UE, and ME deep learning analyses

The analysis begins with preparing the input data using the *prepare_data()* function. This function requires defining the (i) traits, the (ii) input dataset (raw data or relationship matrix), and (iii) environments (in case there are multiple environments). In the case of traits (i), if there is only one trait, a vector containing the phenotypic values is specified by the user. If two or more traits are present, a dataframe is specified by the user, where each column corresponds to an individual trait. In the input datasets (ii), individuals are represented by rows, while columns correspond to SNPs or spectral information, while for the relationship matrix, G- or H-Matrix must be estimated in advance and provided to the system in matrix format. If the data corresponds to multiple environments, the environments (iii) argument is defined as a vector with the name of each of the environments.

### 2.4 Genomic and phenomic prediction using deep learning

Starting from the input data prepared using the *prepare_data()* function and the creation of a list of hyperparameters using the *hyperparameters()* function, a DL model for genomic or phenomic selection can be trained using the *DL_UT()* or *DL_MT()* functions. These functions support model training for UE or ME. Both functions include a test argument, which specifies the desired operation mode:

- Hyperparameter testing (*test=FALSE*): This mode identifies the optimal combination of hyperparameters for model performance. The functions output a data frame containing all possible hyperparameter combinations and their respective efficiency metrics. To analyze these results, the *resume_hyperparameters()* function can be used. This function performs an analysis of variance for each hyperparameter, determines significant differences, and provides a ranked list of the best combinations. Additionally, it generates a bar graph that visually highlights the significance of these differences.

- Final model evaluation (*test=TRUE*): In this mode, the final model is evaluated using a test dataset. This approach is used for both assessing the model’s predictive ability and predicting phenotypic values for new individuals, including the application of the predictive model to unknown field data.

### 2.5 Genomic prediction models and cross-validation

#### 2.5.1 MTMEGPS

The *MTMEGPS* uses DL methods to analyze UT and MT, UE and ME data, as described in Mora-Poblete et al. (2023). A neural network architecture with multiple layers was used to predict traits in both datasets. The network consists of an input layer with *n* neurons (number of features in the dataset after *prepare_data()* function). Three fully connected hidden layers were included, each initialized with approximately two-thirds of the number of neurons of the input layer according to Karsoliya (2012), and this configuration cannot be modified by the user. The output layer has one neuron for UT predictions and *n* neurons (number of traits analyzed) for MT predictions. The neurons in the network are fully connected, where the contribution of each neuron to the overall output is determined by the strength of its connection weights. To improve generalization and mitigate overfitting, a dropout regularization was applied, randomly deactivating 30% of the neurons and their connections during training (this configuration cannot be modified by the user).

The input variables for the MT and ME approach were constructed by concatenating data across environments. In the case of the relationship matrix, this concatenation was performed using the Cholesky decomposition of the genomic relationship matrix and the genotype × environment interaction (G_relationship_×E). In contrast, for the raw data, the Cholesky decomposition was not applied, and the G_raw data_×E interaction was handled directly. To achieve this, design matrices were created for environments (Z_E_), genotypes (Z_G_), and the genotype × environment interaction (Z_GE_). For the relationship matrix case, the genotype design matrix was post-multiplied by the transpose of the upper triangular matrix obtained from the Cholesky decomposition (Q^t^), resulting in Z_G_ = Z_G_Q^t^. In the raw data case, the genotype matrix was post-multiplied by the raw data matrix (R), yielding Z_G_ = Z_G_R. The G×E term was then calculated by post-multiplying the G×E design matrix by the Kronecker product of an identity matrix (of order equal to the number of environments). Finally, the input matrix of covariates used for implementing the DL models was defined as X = [Z_E_, Z_G_, Z_GE_]. It is worth noting that the Bayesian Genomic Linear Regression approach follows a similar implementation for MT and ME analyses.

#### 2.5.2 Bayesian genomic linear regression

For the analysis based on relationship matrices (genomic G and phenomic H), we used the following linear mixed model:


$$\text{y}=1\mu +{\text{Z}}_{\text{env}}\beta_{\text{env}}+{\text{Z}}_{\text{g}}\text{g}+{\text{Z}}_{ge}g_{ge}+\epsilon$$

where y is the n×1 vector of phenotypic records stacked across environments and genotypes, μ is the overall intercept, ${\text{Z}}_{\text{env}}$ is the incidence matrix for environment effects with $\beta_{\text{env}}$ the corresponding vector of fixed site effects, ${\text{Z}}_{g}$ is the incidence matrix linking records to the vector of additive genomic effects g, and ${\text{Z}}_{ge}$ is the incidence matrix for the genotype × environment interaction effects $g_{\text{g}e}$. The residual term is denoted by ϵ. The random effects were assumed to follow:


$$\text{g}∼N(0,\sigma_{g}^{2}\text{G}),{\text{g}}_{ge}∼N(0,\sigma_{ge}^{2}(\text{G}⊗{\text{I}}_{e})),\epsilon ∼N(0,\sigma_{\epsilon }^{2}{\text{I}}_{n})$$

where G is the genomic (or phenomic) relationship matrix, ${\text{I}}_{e}$ is an identity matrix of order equal to the number of environments, and ${\text{I}}_{n}$ is an identity matrix of order equal to the number of observations. These structures correspond to standard multi-environment GBLUP models widely used in breeding programs and were implemented in the BGLR package.

For analysis based on raw data (SNP markers and spectral information), we used the following multivariate linear mixed model:


$$\text{Y}=1\mu^{⊤}+\text{XB}+\text{U}+\text{E}$$

where Y is the n×t phenotype matrix (individuals in rows and traits in columns, with t=1 for UT), μ is the t×1 vector of trait-specific intercepts, X is the incidence matrix for fixed effects, B is the matrix of fixed-effect coefficients, U is the matrix of random effects associated with the predictors (SNPs or spectral covariates), and E is the matrix of residuals. Random effects were assumed to follow:


$$\text{U}∼N(0,\text{K}⊗\Sigma_{u}),\text{E}∼N(0,{\text{I}}_{n}⊗\Sigma_{e})$$

where K is the covariance matrix among predictors, $\Sigma_{u}$ is the trait covariance matrix for random effects, and $\Sigma_{e}$ is the residual covariance matrix. All vectors and matrices are written in boldface, while subscripts are used only to distinguish specific effects (e.g., environments or traits).

#### 2.5.3 DeepGS

The DeepGS model uses convolutional neural network architecture consisting of an input layer, a convolutional layer, a pooling layer, three dropout layers, two fully connected layers, and an output layer (Ma et al., 2017). The input layer receives genotypic markers in an n × m matrix, where n is the number of individuals and m is the number of genotypic markers. The convolutional layer applies eight filters to the input matrix, followed by a max-pooling layer. The output of the pooling layer is passed through a dropout layer with a rate of 0.2 to reduce overfitting. Later, a fully connected layer integrates the features extracted by the convolution, using a dropout rate of 0.1. A nonlinear activation function ReLU is applied in both the convolutional layer and the first fully connected layer. The output from the first fully connected layer is passed to a second fully connected layer with a single neuron and a dropout rate of 0.05. Finally, a linear regression model connects this output to the final output layer, which predicts the phenotypic value of the individual under analysis.

#### 2.5.4 Sommer

The *sommer* package (Covarrubias-Pazaran, 2016) was used to fit univariate and multivariate GBLUP models following the mixed-model framework commonly applied in operational breeding programs. All models were expressed using a unified vector–matrix notation to ensure consistency with the other methods described in this study. For univariate analyses, the following linear mixed model was used:


$$\text{y}=1\mu +{\text{Z}}_{\text{env}}\beta_{\text{env}}+{\text{Z}}_{g}\text{u}+\epsilon$$

where y is the vector of phenotypic records, ${\text{Z}}_{\text{env}}$ and ${\text{Z}}_{g}$ are the design matrices for environment effects and additive genetic effects, respectively, u is the vector of genomic effects, and ϵis the residual vector. Random effects were assumed as:


$$\text{u}∼N(0,\;\sigma_{u}^{2}\text{G}),\epsilon ∼N(0,\;\sigma_{\epsilon }^{2}{\text{I}}_{n})$$

where G is the genomic (or phenomic) relationship matrix.

For UTME analyses, genotype × environment interaction was included:


$$\text{y}=1\mu +{\text{Z}}_{\text{env}}\beta_{\text{env}}+{\text{Z}}_{g}u+{\text{Z}}_{ge}{\text{u}}_{ge}+\epsilon ,$$

$${\text{u}}_{ge}∼N(0,\;\sigma_{ge}^{2}(\text{G}⊗{\text{I}}_{e}))$$

with ${\text{I}}_{e}$ the identity matrix of dimension equal to the number of environments.

For multivariate analyses without explicit environmental effects, the following model was used:


$$\text{Y}=1\mu^{⊤}+{\text{Z}}_{g}\text{U}+\text{E}$$

where Y is the phenotype matrix (individuals × traits), μ is the vector of trait intercepts, U is the matrix of additive genetic effects, and E is the residual matrix. Random components followed:


$$\text{U}∼N(0,\;\text{G}⊗\Sigma_{u}),\text{E}∼N(0,\;{\text{I}}_{n}⊗\Sigma_{e})$$

where $\Sigma_{u}$ is an unstructured trait covariance matrix and $\Sigma_{e}$ is a diagonal residual covariance matrix.

For multi-trait, multi-environment analyses, the following model was used:


$$\text{Y}=1\mu^{⊤}+{\text{Z}}_{g}{\text{U}}_{g}+{\text{Z}}_{\text{env}}{\text{U}}_{\text{env}}+{\text{Z}}_{ge}{\text{U}}_{ge}+\text{E}$$

The random structures were:


$${\text{U}}_{g}∼N(0,\;\text{G}⊗\Sigma_{g}),$$

$${\text{U}}_{\text{env}}∼N(0,\;{\text{I}}_{e}⊗\Sigma_{\text{env}}),$$

$${\text{U}}_{ge}∼N(0,\;(\text{G}⊗{\text{I}}_{e})⊗\Sigma_{ge}),$$

$$\text{E}∼N(0,\;{\text{I}}_{n}⊗\Sigma_{e})$$

where $\Sigma_{g}$ is an unstructured covariance matrix among traits, and $\Sigma_{\text{env}},\;\Sigma_{ge},\;\Sigma_{e}$ are diagonal matrices with trait-specific variances.

#### 2.5.5 bWGR

The *bWGR* package (Xavier et al., 2020) was used to fit all four approaches within a unified Bayesian multivariate GBLUP framework using the *MRR3F()* function. This algorithm implements a factor-analytic multivariate model (Xavier and Habier, 2022; Xavier et al., 2025), enabling flexible estimation of unstructured covariance matrices across traits, environments, or their combinations.

For the UTUE and UTME analyses, genomic effects were modeled using the standard mixed model already described in this study:


$$\text{y}=\text{X}\beta +{\text{Z}}_{g}\text{u}+\epsilon$$

where fixed and random components follow the same assumptions defined for the univariate GBLUP models.

In *bWGR*, these univariate structures are obtained by specifying a single-response vector in **y**, and the MRR3F() algorithm estimates the additive effects using Bayesian shrinkage over the genomic relationship matrix G.

For the MTUE model, the response was arranged as a phenotype matrix Y (individuals × traits). The multivariate model is:


$$\text{Y}=\text{X}\beta +{\text{Z}}_{g}\text{U}+\text{E}$$

with vectorized random components:


$$\text{U}∼N(0,\;\text{G}⊗\Sigma_{g}),\text{E}∼N(0,\;{\text{I}}_{n}⊗\Sigma_{e})$$

where $\Sigma_{g}$ is an unstructured genetic covariance matrix and $\Sigma_{e}$ is a diagonal residual covariance matrix. These covariance structures are estimated automatically through the factor-analytic priors implemented in *bWGR*.

For the MTME analyses, **Y** was arranged so that each column represented a specific trait–environment combination. Under this representation, additive genetic effects, site effects, and genotype × environment interactions are naturally accommodated within the expanded multivariate covariance structure. The random components follow:


$${\text{U}}_{g}∼N(0,\;\text{G}⊗\Sigma_{g}),$$

$${\text{U}}_{\text{env}}∼N(0,\;{\text{I}}_{e}⊗\Sigma_{\text{env}}),$$

$${\text{U}}_{ge}∼N(0,\;(\text{G}⊗{\text{I}}_{e})⊗\Sigma_{ge}),$$

$$\text{E}∼N(0,\;{\text{I}}_{n}⊗\Sigma_{e})$$

where the Σ matrices correspond to trait-specific covariance structures (unstructured for genetic effects, diagonal for environment and residual components).

All analyses were performed in R (R Core Team, 2016) on a workstation running Ubuntu 24.10 equipped with an Intel^®^ Core™ i9-9900KF CPU (16 logical cores) and 61 GB of RAM.

#### 2.5.6 Cross validation

Genomic prediction models were evaluated using 50 cycles of cross-validation. In each cycle, independent and non-overlapping training (80%) and testing (20%) subsets were randomly generated, ensuring complete separation between the data used for model fitting and the data used for validation. Prediction ability was quantified as the Pearson correlation between observed and predicted phenotypes in the testing set for each cycle. Significant differences among models within each approach were evaluated using analysis of variance followed by Tukey’s *post hoc* test (p<0.05). Predictive performance was additionally assessed using the mean squared error of prediction:


$$MSE=\frac{1}{n}\sum {\left(y_{i}-\hat{y_{i}}\right)}^{2}$$

where $y_{i}$ and $\hat{y_{i}}$ represent the observed and predicted values for each sample, respectively.

It is acknowledged that non-linear parameterizations, such as Gaussian kernels within an RKHS framework, could also serve as alternative baseline models. These approaches can reduce discrepancies in model complexity between linear and non-linear methods and therefore represent a potentially more comparable benchmark.

## 3 Results

### 3.1 Software validation and demonstration with internal datasets

#### 3.1.1 Comparison of prediction approaches and hyperparameter optimization

To demonstrate the potential of *MTMEGPS*, two publicly available datasets were utilized: a Maize dataset containing molecular marker information (Mora-Poblete et al., 2023), and an Eucalyptus dataset containing spectral data (Mieres-Castro et al., 2024). The Maize dataset was analyzed using all four approaches (UTUE, UTME, MTUE, and MTME), while the Eucalyptus dataset was evaluated using only the UTUE and MTUE approaches. Both datasets exhibited distinct differences among approaches, with no consistent trend of superiority for any particular method (**Supplementary Table 1**). In the case of the Eucalyptus dataset, the MTUE approach achieved slightly lower predictive ability (0.679) compared with the average UTUE traits (0.684), calculated as the mean of T1 (0.731), T2 (0.654), and T3 (0.668). In contrast, the Maize dataset achieved its highest prediction ability with the UTUE (0.757) approach in environment 1 (**Supplementary Table 1**).

**Figure 2:**
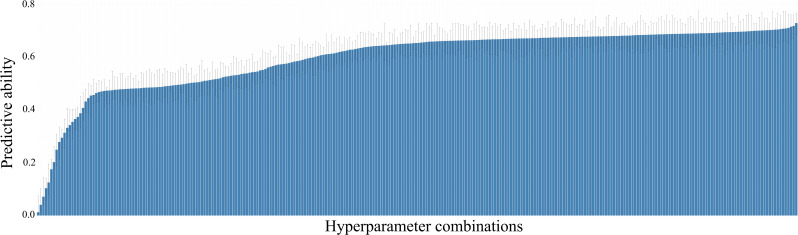
Predictive ability of the UTUE model for Trait 1 across different hyperparameter combinations in the Eucalyptus dataset. Bars represent standard errors.

**Figure 3:**
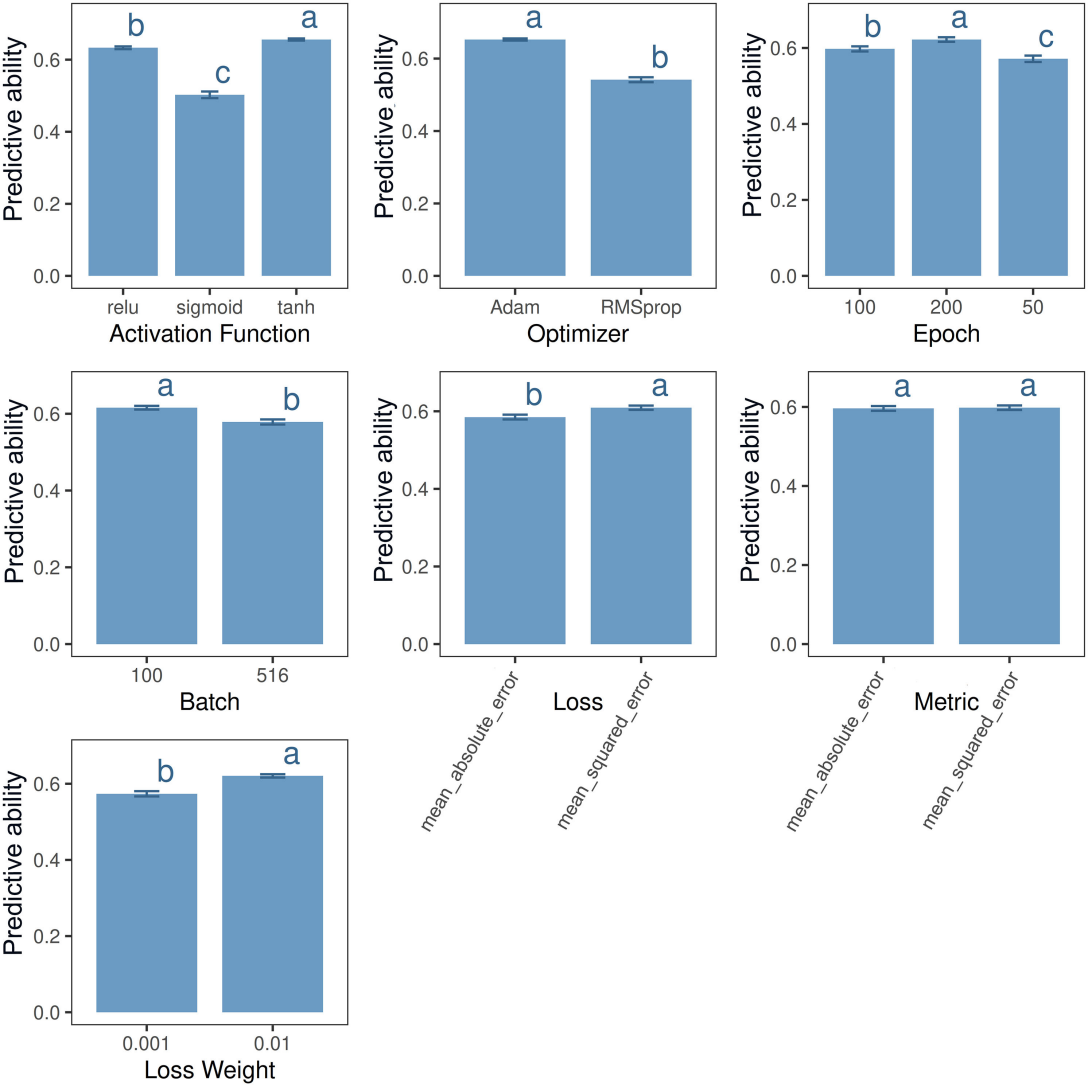
Impact of hyperparameters on the predictive ability of the UTUE model (Trait 1) in the Eucalyptus dataset generated with the *MTMEGPS* package. Different letters indicate significant differences among groups (Tukey *post hoc* test, p < 0.05). Bars represent standard errors.

Hyperparameter tuning played a key role in improving model accuracy. As shown in **Figure 2**, the predictive ability of the UTUE models for the Eucalyptus dataset ranged from 0.012 to 0.729 across 288 hyperparameter combinations (3 activation functions × 2 optimizers × 3 epoch settings × 2 batch sizes × 2 loss functions × 2 evaluation metrics × 2 loss weights). The most effective configuration was consistent across all Eucalyptus models: tanh activation function, Adam optimizer, 100 epochs, mean_squared_error loss function, and a loss weight of 0.01 (see **Figure 3** for further details). A similar pattern was observed regarding optimal hyperparameter settings in the Maize dataset. The best-performing Maize models also consistently used tanh or ReLU activation functions, the Adam optimizer, 100–200 epochs, and a loss weight of 0.01. These findings align with previous studies on genomic and phenomic prediction using DL (Mora-Poblete et al., 2023; Mieres-Castro et al., 2024).

#### 3.1.2 Comparison of approaches in terms of prediction ability and identification of the best hyperparameter combinations

**Table 1** presents the predictive ability and MSE estimates for traits in Eucalyptus using the UTUE and MTUE approaches and for Maize across two environments employing all approaches (UTUE, UTME, MTUE, and MTME). In contrast to the analysis of optimal hyperparameter combinations, the optimized prediction models revealed that, for the Eucalyptus dataset, the MTUE approach achieved slightly higher prediction ability values than the UTUE approach in the *MTMEGPS* model, though the differences were not statistically significant.

**Table 1:** Mean prediction ability of UT and MT and UE and ME approaches using *MTMEGPS*, Bayesian (BGLR package), DeepGS, Sommer and bWGR models for predicting phenotypic traits in Maize and Eucalyptus.

Database	Approach	Trait	Environment	Prediction accuracy				
				MTMEGPS	BGLR	DeepGS	Sommer	bWGR
Eucalyptus	UTUE	T1	–	0.73 (<0.01) A	0.67 (0.01) B	0.44 (0.01) C	0.58 (<0.01) B	0.34 (0.02) D
		T2		0.66 (17.85) A	0.59 (15.67) B	0.57 (8.78) B	0.52 (10.8) B	0.39 (23.51) C
		T3		0.67 (<0.01) A	0.6 (<0.01) B	0.31 (<0.01) C	0.61 (<0.01) B	0.37 (<0.01) C
	MTUE	T1	–	0.72 (<0.01) A	0.67 (<0.01) A	–	0.63 (0.01) B	0.35 (0.02) C
		T2		0.67 (3.11) A	0.59 (17) B	–	0.69 (6.84) A	0.4 (17.08) C
		T3		0.68 (<0.01) A	0.56 (<0.01) B	–	0.59 (<0.01) B	0.38 (<0.01) C
Maize	UTUE	T1	E1	0.76 (8.72) A	0.66 (13.18) B	0.37 (16.88) C	0.71 (2.32) B	0.65 (1.35) B
		T2		0.84 (2.95) A	0.79 (3.79) B	0.41 (18.37) C	0.7 (8.87) B	0.77 (1.2) B
		T3		0.67 (2.57) A	0.62 (3.07) B	0.35 (1.94) C	0.55 (1.74) B	0.52 (3.81) B
		T1	E2	0.62 (1.28) A	0.46 (5.76) B	0.37 (6.29) C	0.42 (11.16) B	0.48 (0.8) B
		T2		0.73 (6.97) A	0.61 (9.94) B	0.28 (6.28) D	0.53 (16.29) CD	0.59 (4.48) BC
		T3		0.58 (0.66) A	0.41 (2.21) B	0.2 (1.92) C	0.48 (0.77) B	0.38 (2.27) B
	UTME	T1	–	0.59 (1.51) A	0.54 (9.77) A	–	0.55 (3.2) B	0.43 (1.87) C
		T2		0.69 (2.31) A	0.67 (4.14) A	–	0.71 (8) A	0.56 (18.12) B
		T3		0.52 (1.41) A	0.44 (2.56) A	–	0.44 (2.14) B	0.27 (1.02) C
	MTUE	T1	E1	0.72 (0.5) A	0.67 (7.52) A	–	0.68 (9.6) A	0.66 (10.51) A
		T2		0.81 (0.51) A	0.72 (0.55) B	–	0.69 (7.23) B	0.78 (1.79) A
		T3		0.62 (0.22) A	0.47 (0.26) B	–	0.52 (2.65) B	0.5 (1.25) B
		T1	E2	0.67 (7.6) A	0.34 (13.26) C	–	0.32 (9.32) C	0.49 (6.12) B
		T2		0.68 (11.03) A	0.53 (16.86) C	–	0.49 (17.82) C	0.61 (17.6) B
		T3		0.39 (3.29) A	0.3 (4.78) B	–	0.39 (2.81) A	0.34 (4.4) A
	MTME	T1	–	0.68 (7.8) A	0.58 (4.43) B	–	0.58 (7.52) B	0.6 (12.6) B
		T2		0.81 (8.01) A	0.53 (5.75) C	–	0.58 (9.13) C	0.71 (6.82) B
		T3		0.42 (2.45) A	0.39 (0.8) BC	–	0.35 (2.53) C	0.43 (3.81) A

Values in parentheses indicate MSE values. Different letters denote significant differences among *MTMEGPS*, Bayesian, DeepGS, Sommer and bWGR models in relation to prediction ability (Tukey *post-hoc* test, p < 0.05).

Prediction abilities ranged from 0.298 (T3 in the MTUE; Environment 2) to 0.794 (T2 in the UTUE; Environment 1) in Bayesian model, and from 0.385 (T3 in the MTUE; Environment 2) to 0.844 (T2 in the UTUE; Environment 1) when *MTMEGPS* model was used. In contrast, the DeepGS model consistently showed the lowest predictive ability under the UTUE approach (MT and ME analyses were not implemented in DeepGS given that this model does not support these types of analyses). The only exception was T2 in Eucalyptus, where DeepGS achieved performance comparable to the Bayesian and Sommer models. Across all scenarios, the *MTMEGPS* model consistently outperformed the Bayesian, DeepGS, Sommer and bWGR models in terms of prediction ability. Specifically, in the Eucalyptus dataset, the UT *MTMEGPS* model yielded prediction ability that were 9.3% (T1) to 13.1% (T3) higher than those of the Bayesian model, 15.8% (T2) to 120.7% (T3) higher than those of the DeepGS model, 11.2% (T3) to 26.3% (T1) in Sommer model, and 67.1% (T2) to 114.1% (T1) in bWGR model. In the MT analysis, improvements show a similar trend across all models; however, in T2, Sommer exceeds MTMEGPS by 3.5%. Similarly, for the Maize dataset, the MTMEGPS models outperformed all other models (Bayesian, DeepGS, Sommer, and bWGR) across all evaluated scenarios, except in T2 UTME (Sommer), T3 MTUE (Sommer), and T3 MTME (bWGR). Interestingly, unlike in Eucalyptus, the average prediction ability for UTUE approach was higher than MTUE, in all models (*MTMEGPS*, Bayesian, Sommer and bWGR).

In terms of MSE, a clear inverse relationship was observed with predictive ability, confirming that higher predictive correlations were consistently associated with lower error values. Although MTMEGPS exhibited higher predictive ability compared with the other models, in the Eucalyptus dataset (under UTUE approach), the Bayesian, DeepGS, and Sommer models achieved the lowest MSE values for the T2 trait, while the remaining models showed comparable performance. In contrast, for the Maize dataset, the MTMEGPS model consistently produced the lowest prediction errors across most evaluated scenarios (38%), followed by bWGR (25%), BGLR (21%), Sommer (13%), and DeepGS (4%). This performance advantage became more pronounced under the MT approach, where MTMEGPS outperformed the other models in 44% of scenarios. Moreover, averaged across all maize scenarios, MTMEGPS achieved reductions in mean MSE compared with all other models, underscoring its superior efficiency in minimizing prediction errors.

Overall, the results indicate that no single approach consistently delivers the highest prediction ability or reduction of MSE. Thus, it is essential to evaluate all approaches to identify the most suitable predictive model for a given dataset. Furthermore, *MTMEGPS* models consistently demonstrated superior performance across most approaches, traits, and environments, underscoring their better prediction ability and MSE compared to Bayesian, Sommer and DeepGS models.

### 3.2 Software validation using an independent multi-environment maize dataset

**Figure 4:**
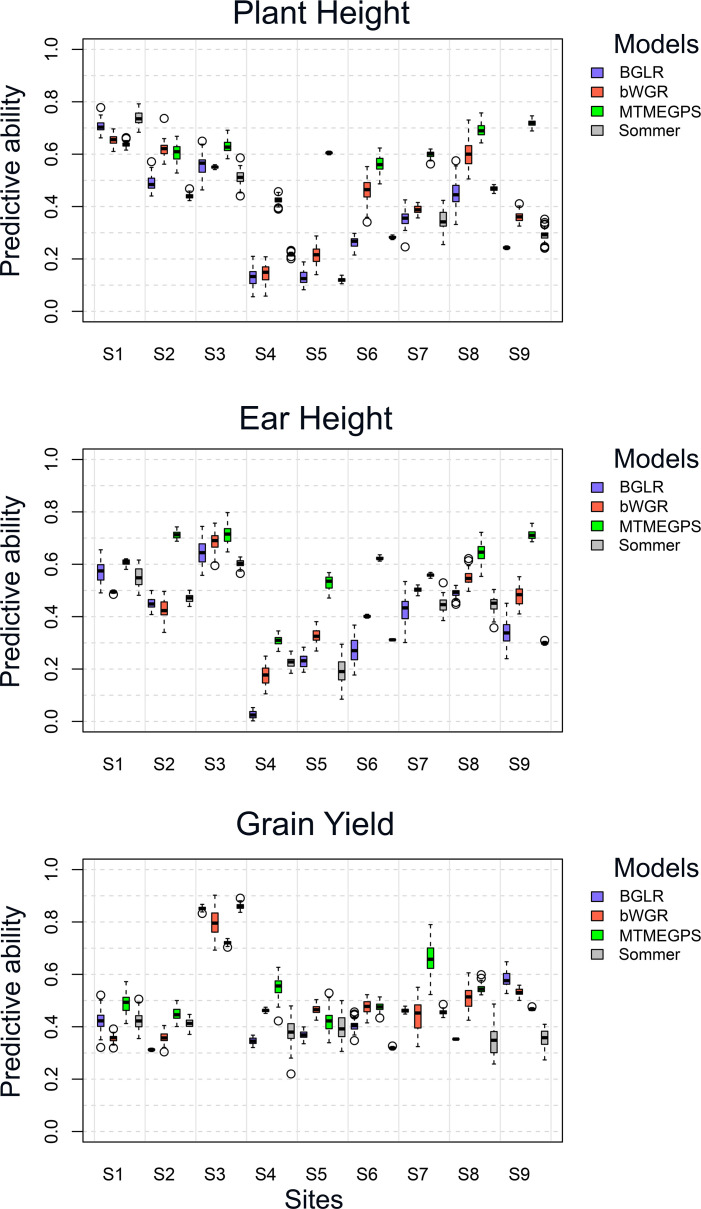
Boxplots of predictive ability for the *MTMEGPS*, BGLR, Sommer, and bWGR models under the MTME approach for the three traits (Plant Height, Ear Height, and Grain Yield) across all sites.

**Supplementary Tables 2**, **3**; **Figure 4** showed the consistent superiority of the *MTMEGPS* model in terms of prediction ability. Across all approaches, *MTMEGPS* achieved the highest predictive performance, particularly under the more complex MTME and MTUE frameworks. Considering all 36 trait–site–approach combinations (3 traits × 9 sites × 4 approaches), *MTMEGPS* obtained the highest predictive ability in 76% of the scenarios (**Supplementary Tables 2**, **3**). Specifically, under the MTME approach, *MTMEGPS* achieved the highest predictive ability for Grain Yield in 6 out of 9 sites (S1, S2, S4, S6, S7, and S8) and for Plant Height in 7 out of 9 sites (S3, S4, S5, S6, S7, S8, and S9). This superiority of *MTMEGPS* over other models is further illustrated in **Figure 4**, where *MTMEGPS* outperformed both BGLR, Sommer and bWGR models across most scenarios. Similarly, under the MTUE approach, *MTMEGPS* showed the best predictive ability in 81% of the scenarios, with 56% of them being significantly higher than the other models. Moreover, under UTME and UTUE, *MTMEGPS* also outperformed the competing models in 70% scenarios. On the other hand, in terms of MSE, *MTMEGPS* achieved the lowest values primarily under the MTUE and UTUE approaches (41% and 33% of the scenarios, respectively). In contrast, BGLR showed the lowest MSE values in most MTME (37%) and UTME (44%) approaches. Overall, these results demonstrate that, in this independent maize dataset, the *MTMEGPS* model consistently outperformed the other models in terms of predictive ability, while exhibiting moderate performance with respect to MSE. This finding suggests that *MTMEGPS* effectively captures inter-trait relationships that contribute to enhanced prediction ability, even when minor trade-offs in error magnitude are present.

## 4 Discussion

We developed *MTMEGPS*, the first R package that provides a streamlined end-to-end workflow (**Figure 1**) for genomic and phenomic selection using molecular markers and NIR spectral data, respectively. Our user-friendly and comprehensive workflow supports UT and MT analyses, as well as UE and ME evaluations, using a DL model. Additionally, *MTMEGPS* incorporates automated hyperparameter tuning and searching to optimize prediction ability. This study presents an end-to-end example of the MTMEGPS workflow for genomic and phenomic selection in plants and benchmarks its performance against Bayesian models and DeepGS. To illustrate its capabilities, we analyzed two datasets: a molecular marker dataset for Maize (Mora-Poblete et al., 2023) and an NIR spectral dataset for Eucalyptus (Mieres-Castro et al., 2024).

Hyperparameter tuning is a central feature of the *MTMEGPS* package. As illustrated in **Figure 2**, different hyperparameter combinations can yield prediction ability ranging from 0.012 to 0.729, underscoring the critical importance of identifying the optimal configuration. In this sense, Ilemobayo et al. (2024) emphasized that effective hyperparameter optimization is essential for improving model performance and generalization. Well-tuned hyperparameters can lead to substantial gains in predictive ability, whereas poorly selected values often result in suboptimal models. The *MTMEGPS* package is the first of its kind to offer a streamlined comparison of multiple hyperparameter configurations through a parallelized architecture optimized for both CPU and GPU, reducing computation time. Additionally, the package generates detailed summaries of all tested hyperparameters (**Figure 3**), enhancing user interpretation and facilitating informed decision-making.

The use of the *MTMEGPS* model with the optimal hyperparameter combination consistently resulted in higher prediction ability compared to the Bayesian, Sommer and bWGR models across all tested scenarios (UTUE, UTME, MTUE, and MTME) and for all traits. Similarly, *MTMEGPS* also outperformed DeepGS under the UTUE approach for every trait evaluated. These results suggest that *MTMEGPS* can be a valuable tool for selecting superior individuals based on genomic or spectral information, achieving between 2% and 97% superiority over its counterpart Bayesian model (**Table 1**), 16% and 199% of DeepGS, 6.7% and 109% of Sommer (except in: MTUE-T2-Eucalyptus, UTME-T2-Maize, and MTUE-T3-Environment 2-Maize), and 16% and 199% in bWGR (except in MTME-T3-Maize). It should be noted that although the package was trained and tested on Maize and Eucalyptus, it can be applied to various vegetal/animal species as well. These findings align with previous studies emphasizing the advantages of DL for improving the prediction ability of complex traits (Montesinos-López et al., 2018; Mora-Poblete et al., 2023; Mieres-Castro et al., 2024). On the other hand, it is important to note that in the Maize dataset, the MT approach enhanced prediction ability compared to the UT approach, while in the Eucalyptus dataset, the UT model outperformed the MT approach. These differences underscore the importance of selecting the appropriate modeling strategy to maximize prediction ability and accelerate genetic improvement in different species. Consistent with these findings, results presented in **Supplementary Tables 2**, **3**; **Figure 4** further confirm the superior performance of the *MTMEGPS* model in terms of predictive ability, particularly under the more complex MTME and MTUE frameworks. However, regarding MSE, *MTMEGPS* exhibited moderate performance compared with the other models, indicating that while it consistently attained higher predictive ability, this advantage was accompanied by intermediate error magnitudes. It is important to note that model performance should be assessed through multiple complementary metrics, as no single indicator can fully capture predictive quality. When a model demonstrates superior predictive ability but moderate MSE values, its practical superiority lies primarily in its capacity to model complexity, nonlinear relationships among traits, even at the cost of slightly higher MSE. This suggests that *MTMEGPS* achieves provides a favorable balance between predictive accuracy and error control, a pattern consistent with other studies (Ahmed et al., 2024).

Overall, our results underscore the potential of *MTMEGPS* as a powerful tool for breeding applications, particularly due to its ability to model complex trait interactions without requiring prior biological knowledge. Notably, *MTMEGPS* is the first package to integrate both molecular and NIR spectral data for genomic and phenomic selection using a DL framework optimized with parallel computing, significantly reducing analysis time. In this regard, MTMEGPS required only 6.3 seconds to run a UTUE model with one repetition per trait, a runtime comparable to Sommer (6.06 seconds), though higher than bWGR (2 seconds). Nevertheless, all three models were substantially faster than the Bayesian model, which required 3 minutes and 1.6 seconds, and DeepGS, which required 48.9 seconds. This efficiency allows for the early identification of superior individuals, which can accelerate breeding cycles, improve selection accuracy, and support effective management of genetic diversity (Grattapaglia, 2017), all while maintaining computational efficiency suitable for breeding programs. It is important to note that *MTMEGPS* allows the use of only one relationship matrix per analysis; therefore, it is not possible to include multiple matrices for additive, dominance, and epistatic effects simultaneously. However, users can create a combined matrix that integrates additive, dominance, and epistatic effects into a single relationship matrix, which can then be directly loaded into the *DL_UT()* and *DL_MT()* functions of the *MTMEGPS* package. Given the promising results of this study, we hope that the breeding science community will adopt *MTMEGPS* and expand its applications to other plant and animal species.

## Data Availability

The original contributions presented in the study are included in the article/**Supplementary Material**. Further inquiries can be directed to the corresponding author.
