# Supplementary material for: MTMEGPS: An R package for multi-trait and multi-environment genomic and phenomic selection using deep learning
Source: Front Plant Sci. 2026 Jan 5;16:1674985. doi: 10.3389/fpls.2025.1674985 (PMC12812867; doi:10.3389/fpls.2025.1674985)
Supplement: Supplementary file 1 [file SupplementaryFile1.pdf]

frontiers

Supplementary Material

1 Supplementary Tables

Supplementary Table 1. Mean prediction ability of Uni- and Multi-Trait and Uni- and Multi-Environment Deep Learning models for predicting phenotypic traits in Maize and Eucalyptus.

	Approach	Trait	Environment	Prediction Ability	Standard Error
Eucalyptus	UTUE	T1	E1	0.731	0.010
		T2	E1	0.654	0.024
		T3	E1	0.668	0.018
	MTUE	All	-	0.679	0.007
Maize	UTUE	T1	E1	0.758	0.013
		T2		0.846	0.012
		T3		0.666	0.033
		T1	E2	0.618	0.049
		T2		0.729	0.008
		T3		0.583	0.011
	UTME	T1	-	0.581	0.008
		T2		0.68	0.007
		T3		0.523	0.030
	MTUE	All	E1	0.712	0.044
		All	E2	0.57	0.046
	MTME	All	-	0.64	0.060

Supplementary Table 2. Mean prediction ability of UTUE and UTME MTMEGPS, BGLR, DeepGS, Sommer and bWGR models for predicting Plant Height, Ear Height, and Grain Yield traits, across all sites.

Trait	Site	UTME				UTUE				
		MTMEGPS	BGLR	Sommer	bWGR	MTMEGPS	BGLR	DeepGS	Sommer	bWGR
Plant Height	S1	0.57 (52.9) c	0.84 (8.2) a	0.62 (27.3) bc	0.63 (60.5) b	0.64 (7.4) c	0.98 (4.4) a	0.66 (9.3) c	0.69 (21.2) c	0.77 (7.5) b
Ear Height		0.62 (5.3) b	0.67 (21.7) a	0.46 (27.8) c	0.65 (15.5) ab	0.73 (4.3) a	0.74 (34.7) a	0.6 (5.1) b	0.6 (19.2) b	0.76 (14.6) a
Grain Yield		0.49 (62.8) a	0.38 (9.1) b	0.4 (72.9) b	0.36 (125) b	0.59 (68.4) a	0.41 (152.1) c	0.46 (37.5) bc	0.61 (59.9) a	0.48 (85.7) b
Plant Height	S2	0.69 (35.8) a	0.57 (17.7) b	0.49 (42.8) c	0.56 (60.4) b	0.86 (13.1) a	0.6 (7) b	0.49 (10.1) c	0.5 (34.7) c	0.58 (10.6) b
Ear Height		0.74 (18) a	0.45 (32.9) b	0.51 (6) b	0.49 (31.6) b	0.82 (2.2) a	0.47 (16.1) c	0.47 (3.4) c	0.6 (23.9) b	0.58 (3) b
Grain Yield		0.46 (31.1) a	0.25 (16.5) b	0.43 (80.6) a	0.3 (15.6) b	0.56 (12) a	0.4 (15.2) b	0.32 (42.3) c	0.51 (19.3) a	0.54 (31.9) a
Plant Height	S3	0.45 (61) c	0.53 (32.2) b	0.63 (36.3) a	0.46 (60.5) c	0.53 (38.1) b	0.48 (37.8) c	0.47 (53.3) c	0.66 (19.4) a	0.55 (25.1) b
Ear Height		0.66 (35.8) a	0.55 (10.5) b	0.58 (25.7) b	0.68 (35.4) a	0.63 (5.1) a	0.58 (29.7) ab	0.52 (4.5) b	0.63 (10.8) a	0.57 (16.6) ab
Grain Yield		0.88 (62.5) a	0.84 (40.1) b	0.85 (28.4) ab	0.77 (171.2) c	0.82 (17.9) b	0.82 (135.8) b	0.65 (41.7) c	0.87 (75.8) b	0.94 (52.1) a
Plant Height	S4	0.45 (76.9) a	0.05 (41.7) c	0.13 (22.9) b	0.15 (35) b	0.43 (2.5) a	0 (94.3) d	0.01 (6.8) d	0.08 (8.7) c	0.13 (2.9) b
Ear Height		0.24 (32.2) a	0.16 (25) b	0.23 (42.6) a	0.24 (30.3) a	0.28 (14.6) a	0.16 (35.6) b	-0.04 (28.5) c	0.19 (41.3) b	0.11 (26.3) b
Grain Yield		0.6 (282.6) a	0.45 (39.1) b	0.5 (143.6) b	0.6 (93.5) a	0.62 (124.4) a	0.44 (421.6) c	0.43 (112.9) c	0.56 (187.9) b	0.6 (132.8) ab
Plant Height	S5	0.61 (53.2) a	0.13 (81.8) c	0.08 (95.8) d	0.3 (125.7) b	0.66 (58) a	0.24 (60.3) c	0.22 (58.3) c	0.13 (69.6) d	0.44 (41.9) b
Ear Height		0.56 (1) a	0.12 (19.1) c	0.24 (4.7) b	0.28 (60.7) b	0.58 (9) a	0.08 (25.6) d	0.17 (2.1) c	0.21 (28.5) bc	0.26 (11.5) b
Grain Yield		0.37 (5.3) b	0.35 (47.7) b	0.45 (142.6) a	0.43 (173.3) a	0.45 (144.8) b	0.36 (380.5) c	0.35 (168.5) c	0.6 (172.8) a	0.44 (152.3) b
Plant Height	S6	0.47 (2.8) a	0.31 (36.8) b	0.26 (49.4) c	0.36 (127.3) b	0.44 (4.5) a	0.38 (60.7) b	0.33 (12.4) c	0.39 (8.2) b	0.39 (4.7) b
Ear Height		0.55 (81.3) a	0.24 (24.8) c	0.43 (62.5) b	0.49 (105.5) a	0.63 (15.9) a	0.17 (6.3) e	0.28 (4.7) d	0.55 (68.5) b	0.43 (20.8) c
Grain Yield		0.53 (28) a	0.25 (234.6) c	0.39 (65.7) b	0.38 (439.2) b	0.52 (160.9) a	0.39 (180.9) b	0.24 (131.1) c	0.38 (200.9) b	0.4 (88.3) b
Plant Height	S7	0.47 (61.6) a	0.39 (47) b	0.27 (110.1) c	0.46 (189.3) a	0.56 (31.3) a	0.38 (60.6) bc	0.34 (58) cd	0.29 (89.6) d	0.44 (28.2) b
Ear Height		0.7 (16.5) a	0.57 (1) b	0.34 (24.3) c	0.52 (0.5) b	0.74 (12.7) a	0.51 (45.9) b	0.43 (7.7) c	0.35 (9.1) d	0.48 (0.6) b
Grain Yield		0.53 (68.7) a	0.41 (75.2) b	0.55 (62.8) a	0.57 (179.6) a	0.55 (36.8) a	0.41 (82.7) b	0.3 (55.1) c	0.55 (17.3) a	0.43 (3.8) b
Plant Height	S8	0.68 (92.5) a	0.45 (42.8) c	0.47 (33.9) c	0.59 (56.2) b	0.62 (31.9) a	0.43 (83.9) b	0.41 (39.7) b	0.61 (19.1) a	0.67 (37.1) a
Ear Height		0.8 (9.2) a	0.68 (18.9) b	0.41 (1.6) c	0.62 (34.9) b	0.89 (10.4) a	0.71 (12.1) b	0.61 (12.7) c	0.46 (9.3) d	0.65 (9.1) bc
Grain Yield		0.62 (41.2) a	0.36 (202.4) d	0.43 (182.9) c	0.53 (174.6) b	0.67 (50.5) a	0.43 (300.2) b	0.33 (37.1) c	0.5 (97.7) b	0.6 (10.5) a
Plant Height	S9	0.72 (50.8) a	0.09 (93.8) c	0.44 (53.5) b	0.41 (40.5) b	0.84 (1.7) a	0.07 (182) d	0.37 (88.2) c	0.51 (26.6) b	0.38 (15.8) c

Ear Height		0.83 (17.1) a	0.45 (6.8) c	0.3 (2.6) d	0.53 (31.7) b	0.99 (9.3) a	0.5 (42) c	0.45 (11.2) c	0.32 (1.7) d	0.64 (28.3) b
Grain Yield		0.32 (287.9) c	0.59 (28.8) a	0.51 (118.4) b	0.57 (173.5) a	0.36 (111.9) c	0.65 (56.4) a	0.42 (91.9) c	0.55 (157.1) b	0.44 (115.5) c

Values in parentheses indicate MSE values. Different letters denote significant differences among MTMEGPS, Bayesian, DeepGS, Sommer and bWGR models in relation to prediction ability (Tukey post-hoc test, $p < 0.05$).

Supplementary Table 3. Mean prediction ability of MTUE and MTME MTMEGPS, BGLR, Sommer and bWGR models for predicting Plant Height, Ear Height, and Grain Yield traits, across all sites.

Trait	Site	MTME				MTUE			
		MTMEGPS	BGLR	Sommer	bWGR	MTMEGPS	BGLR	Sommer	bWGR
Plant Height	S1	0.64 (15.5) b	0.71 (1.9) ab	0.74 (9.7) a	0.66 (59.8) b	0.68 (25.9) b	0.77 (16.4) a	0.66 (24.2) b	0.63 (50.9) b
Ear Height		0.61 (20.8) a	0.57 (4.5) b	0.55 (5.4) b	0.5 (33.9) c	0.67 (32) a	0.61 (1.2) b	0.54 (10.8) c	0.62 (42.5) b
Grain Yield		0.48 (86.7) a	0.43 (92.8) a	0.43 (91.3) a	0.35 (137.2) b	0.58 (32.9) a	0.42 (43.7) c	0.47 (35.1) b	0.58 (93.4) a
Plant Height	S2	0.61 (54.2) a	0.49 (1) b	0.44 (13.7) b	0.62 (53.2) a	0.62 (6) a	0.5 (2.8) c	0.45 (38.2) d	0.55 (18.6) b
Ear Height		0.71 (9) a	0.45 (22.9) b	0.47 (27.5) b	0.43 (34.7) b	0.8 (12.2) a	0.44 (21) b	0.47 (29.8) b	0.5 (4.8) b
Grain Yield		0.45 (9.2) a	0.31 (26.6) c	0.41 (27.4) ab	0.36 (15.8) bc	0.54 (17.1) a	0.27 (90.3) c	0.48 (6.3) ab	0.41 (15.6) b
Plant Height	S3	0.63 (35.2) a	0.56 (26.2) ab	0.51 (5.1) b	0.55 (52.4) ab	0.56 (3) a	0.49 (30.7) b	0.56 (23.4) a	0.43 (31.9) c
Ear Height		0.71 (38.1) a	0.65 (18.7) b	0.6 (0.5) c	0.7 (16.3) a	0.68 (2.1) a	0.64 (2.3) ab	0.61 (9.1) b	0.62 (41.9) b
Grain Yield		0.72 (112.3) b	0.85 (38.2) a	0.86 (21.3) a	0.8 (146.4) a	0.8 (24.8) bc	0.77 (31.2) c	0.93 (20.5) a	0.86 (66.5) b
Plant Height	S4	0.42 (46.5) a	0.14 (37.4) c	0.22 (33.4) b	0.14 (90.9) c	0.41 (59.7) a	0.14 (17.3) c	0.2 (24.9) bc	0.25 (88.1) b
Ear Height		0.31 (69.6) a	0.03 (91.4) c	0.23 (21.7) ab	0.17 (42.8) b	0.27 (21.5) a	0.08 (22.8) c	0.19 (23.2) b	0.12 (23.6) c
Grain Yield		0.56 (10.6) a	0.34 (14) c	0.38 (177.7) c	0.46 (252.8) b	0.53 (313.5) a	0.37 (114.1) b	0.4 (120.7) b	0.35 (211.5) b
Plant Height	S5	0.6 (23.5) a	0.13 (63.1) c	0.12 (69.4) c	0.21 (131.2) b	0.59 (18.4) a	0.13 (18.9) b	0.06 (38.3) c	0.16 (56.1) b
Ear Height		0.54 (28.8) a	0.23 (16.3) c	0.21 (15.7) c	0.33 (40.9) b	0.54 (8.6) a	0.21 (24.1) c	0.32 (5.2) b	0.32 (42.9) b
Grain Yield		0.43 (69.9) ab	0.37 (199.6) b	0.4 (79.2) b	0.47 (374.4) a	0.48 (104.3) a	0.48 (114.4) a	0.38 (251.1) b	0.49 (129.6) a
Plant Height	S6	0.55 (2.6) a	0.27 (10.2) c	0.28 (44) c	0.46 (6) b	0.48 (65.8) a	0.21 (68.8) c	0.28 (8.5) b	0.28 (93.9) b
Ear Height		0.62 (9.6) a	0.27 (31.8) c	0.31 (6) c	0.4 (32) b	0.57 (12.3) a	0.26 (25.9) d	0.34 (68.5) c	0.42 (20.3) b
Grain Yield		0.48 (113.7) a	0.4 (124.5) b	0.32 (197) c	0.47 (471.8) a	0.44 (393.5) a	0.35 (191.2) b	0.44 (260.4) a	0.36 (514.4) b
Plant Height	S7	0.6 (23.4) a	0.35 (29.2) c	0.35 (58.9) c	0.39 (74.5) b	0.55 (71.8) a	0.43 (22.2) b	0.26 (127) c	0.46 (193.4) b
Ear Height		0.56 (13.7) a	0.44 (8) b	0.44 (19.1) b	0.5 (33.1) ab	0.64 (5.7) a	0.52 (9.5) b	0.39 (8.2) c	0.5 (9.7) b
Grain Yield		0.66 (11.4) a	0.46 (54.4) b	0.46 (16.9) b	0.46 (12.7) b	0.58 (7) a	0.47 (73.6) b	0.56 (69.8) a	0.48 (8.3) b
Plant Height	S8	0.7 (56.9) a	0.44 (66.4) c	0.47 (48.6) c	0.61 (70.3) b	0.68 (29.2) a	0.43 (41.2) c	0.52 (16.6) b	0.59 (36.4) b
Ear Height		0.66 (31.7) a	0.49 (23) bc	0.44 (5.6) c	0.55 (16.5) b	0.78 (10.7) a	0.54 (2.8) c	0.43 (5.1) d	0.6 (19.9) b
Grain Yield		0.54 (8.6) a	0.35 (159.2) b	0.35 (93.6) b	0.51 (10.3) a	0.53 (19.6) a	0.35 (113.4) c	0.46 (22.1) ab	0.4 (248.6) bc
Plant Height	S9	0.72 (53.2) a	0.24 (41.6) c	0.29 (86.8) c	0.36 (86.2) b	0.78 (4.5) a	0.14 (54.4) c	0.38 (28.2) b	0.32 (73.4) b
Ear Height		0.72 (5.5) a	0.34 (14.3) c	0.3 (29.6) c	0.47 (5.5) b	0.78 (25.3) a	0.36 (26.8) c	0.25 (13.9) d	0.49 (33.9) b
Grain Yield		0.47 (323.3) c	0.59 (114.4) a	0.35 (98.5) d	0.53 (251.8) b	0.38 (113.6) c	0.65 (63.7) a	0.46 (191.5) b	0.44 (355.8) b

Values in parentheses indicate MSE values. Different letters denote significant differences among MTMEGPS, Bayesian, Sommer and bWGR models in relation to prediction ability (Tukey post-hoc test, $p < 0.05$).

2 Supplementary Codes

Codes.R. Code to run the four approaches of the MTMEGPS package, perform the search for the best hyperparameters, and run cross-validations.

```
library(MTMEGPS)

setwd("path to data folder")

########################
#Prepare datasets
########################

G=read.table("kinship matrix",h=T)
pheno=read.table("phenotype matrix",h=T)
head(pheno)

#Models MTMEGPS

########################
#UTUE
########################
sites=unique(pheno$S)
sites

s=sites[1] #site 1
t=pheno[,1] #phenotype 1 ("Plant Height")

x=prepare_data(pheno[pheno$S==s,],as.matrix(G))
y = pheno[pheno$S==s,t]
dim(x); length(y)

#Identify best hyperparameters combination
#Set hyperparameters to test
act=c("linear","relu","sigmoid")
opt=c("RMSprop","Adam")
ep <- c(50,100,150)
bat <- c(round(length(y)*0.5,0), length(y))
los <- c("mean_absolute_error", "mean_absolute_percentage_error")
met <- c("mean_absolute_error", "mean_absolute_percentage_error")
losw <- c(0.001, 0.01)
rep=3
```

```
hyp=hyperparameters(activations=act,optimizers=opt,epochs=ep,batchs=bat,losses=los,metrics=met,l
oss_weights=losw,repetitions=rep)

#Execute the model UTUE to search the best hyperparameter combination
results_hyperparameters_matrix=NULL
#n iteratios for cross-validation
for(r in 1:iteratios){
 test <- sample(1:nrow(G), round(nrow(G) * 0.8))
 Post_trn=c(test)
 xtr = x[Post_trn,]
 xts = x[-Post_trn,]
 ytr = y[Post_trn]
 yts = y[-Post_trn]
 results_hyperparameters_matrix=rbind(results_hyperparameters_matrix,DL_UT(xtr,txs,ytr,yts,hyp))
}
resume_hyperparameters(results_hyperparameters_matrix,(rep*r))

#Select the best hyperparameters combination
act=c(activation function)
opt=c(optimizer)
ep <- c(epoch)
bat <- c(batch)
los <- c(losses)
met <- c(metrics)
losw <- c(loss_weights)
rep=1
hyp=hyperparameters(activations=act,optimizers=opt,epochs=ep,batchs=bat,losses=los,metrics=met,l
oss_weights=losw,repetitions=rep)

#Execute the model UTUE with best hyperparameter combination. n iterations were used for cross-
validation

result=NULL
for(rep in 1:iterations){
  set.seed(rep)
  test=sample(1:nrow(x),nrow(x)*0.2)
  xtr = x[-test,]
  xts = x[test,]
  ytr = y[-test]
  yts = y[test]
  yhat=DL_UT(x,y,hyp,test=TRUE)
  result=rbind(result,(cor(yhat,yts)))
}
result


#######################
#UTME
```

```
#######################
sites=unique(pheno$S)
sites

x=prepare_data(pheno,as.matrix(G))
y = pheno[,t]
dim(x); length(y)

#Identify best hyperparameters combination
#Set hyperparameters to test
act=c("linear","relu","sigmoid")
opt=c("RMSprop","Adam")
ep <- c(50,100,150)
bat <- c(round(length(y)*0.5,0), length(y))
los <- c("mean_absolute_error", "mean_absolute_percentage_error")
met <- c("mean_absolute_error", "mean_absolute_percentage_error")
losw <- c(0.001, 0.01)
rep=3
hyp=hyperparameters(activations=act,optimizers=opt,epochs=ep,batchs=bat,losses=los,metrics=met,l
oss_weights=losw,repetitions=rep)

results_hyperparameters_matrix=NULL
#n iteratios for cross-validation
for(r in 1:iteratios){
 test <- sample(1:nrow(G), round(nrow(G) * 0.8))
 Post_trn=c(test,test+nrow(G))
 xtr = x[Post_trn,]
 xts = x[-Post_trn,]
 ytr = y[Post_trn]
 yts = y[-Post_trn]
 results_hyperparameters_matrix=rbind(results_hyperparameters_matrix,DL_UT(xtr,txs,ytr,yts,hyp))
}
resume_hyperparameters(results_hyperparameters_matrix,(rep*r))

#Select the best hyperparameters combination
act=c(activation function)
opt=c(optimizer)
ep <- c(epoch)
bat <- c(batch)
los <- c(losses)
met <- c(metrics)
losw <- c(loss_weights)
rep=1
hyp=hyperparameters(activations=act,optimizers=opt,epochs=ep,batchs=bat,losses=los,metrics=met,l
oss_weights=losw,repetitions=rep)
```

```
#Execute the model UTME with best hyperparameter combination. n iterations were used for cross-validation

result=NULL
for(rep in 1:iterations){
  set.seed(rep)
  test=sample(1:nrow(x),nrow(x)*0.2)
  xtr = x[-test,]
  xts = x[test,]
  ytr = y[-test]
  yts = y[test]
  yhat=DL_UT(x,y,hyp,test=TRUE)
  result=rbind(result,(cor(yhat,yts)))
}

########################
#MTME
########################
sites=unique(pheno$S)
sites

x=prepare_data(pheno,as.matrix(G))
y = pheno[,1:3] #all traits (Plant Height, Ear Height, Grain Yield)
dim(x); dim(y)

#Identify best hyperparameters combination
#Set hyperparameters to test
act=c("linear","relu","sigmoid")
opt=c("RMSprop","Adam")
ep <- c(50,100,150)
bat <- c(round(length(y)*0.5,0), length(y))
los <- c("mean_absolute_error", "mean_absolute_percentage_error")
met <- c("mean_absolute_error", "mean_absolute_percentage_error")
losw <- c(0.001, 0.01)
rep=3
hyp=hyperparameters(activations=act,optimizers=opt,epochs=ep,batchs=bat,losses=los,metrics=met,loss_weights=losw,repetitions=rep)

results_hyperparameters_matrix=NULL
#n iteratios for cross-validation
for(r in 1:iteratios){
 test <- sample(1:nrow(G), round(nrow(G) * 0.8))
 Post_trn=c(test,test+nrow(G))
 xtr = x[Post_trn,]
 xts = x[-Post_trn,]
 ytr = y[Post_trn]
 yts = y[-Post_trn]
 results_hyperparameters_matrix=rbind(results_hyperparameters_matrix,DL_MT(xtr,txs,ytr,yts,hyp))
```

```
}
resume_hyperparameters(results_hyperparameters_matrix,(rep*r))

#Select the best hyperparameters combination
act=c(activation function)
opt=c(optimizer)
ep <- c(epoch)
bat <- c(batch)
los <- c(losses)
met <- c(metrics)
losw <- c(loss_weights)
rep=1
hyp=hyperparameters(activations=act,optimizers=opt,epochs=ep,batchs=bat,losses=los,metrics=met,l
oss_weights=losw,repetitions=rep)

#Execute the model MTME with best hyperparameter combination. n iterations were used for cross-
validation

result=NULL
for(rep in 1:iterations){
  set.seed(rep)
  test=sample(1:nrow(x),nrow(x)*0.2)
  xtr = x[-test,]
  xts = x[test,]
  ytr = y[-test,]
  yts = y[test,]
  yhat=DL_MT(x,y,hyp,test=TRUE)
  result=rbind(result,diag(cor(yhat,yts)))
}

#######################
#MTUE
#######################
sites=unique(pheno$S)
sites

s=site[1] #site 1

x=prepare_data(pheno[pheno$S==s,],as.matrix(G))
y = pheno[pheno$S==s,1:3]
dim(x); dim(y)

#Identify best hyperparameters combination
#Set hyperparameters to test
act=c("linear","relu","sigmoid")
opt=c("RMSprop","Adam")
ep <- c(50,100,150)
```

```
bat <- c(round(length(y)*0.5,0), length(y))
los <- c("mean_absolute_error", "mean_absolute_percentage_error")
met <- c("mean_absolute_error", "mean_absolute_percentage_error")
losw <- c(0.001, 0.01)
rep=3
hyp=hyperparameters(activations=act,optimizers=opt,epochs=ep,batchs=bat,losses=los,metrics=met,l
oss_weights=losw,repetitions=rep)

results_hyperparameters_matrix=NULL
#n iteratios for cross-validation
for(r in 1:iteratios){
 test <- sample(1:nrow(G), round(nrow(G) * 0.8))
 Post_trn=c(test)
 xtr = x[Post_trn,]
 xts = x[-Post_trn,]
 ytr = y[Post_trn]
 yts = y[-Post_trn]
 results_hyperparameters_matrix=rbind(results_hyperparameters_matrix,DL_MT(xtr,txs,ytr,yts,hyp))
}
resume_hyperparameters(results_hyperparameters_matrix,(rep*r))

#Select the best hyperparameters combination
act=c(activation function)
opt=c(optimizer)
ep <- c(epoch)
bat <- c(batch)
los <- c(losses)
met <- c(metrics)
losw <- c(loss_weights)
rep=1
hyp=hyperparameters(activations=act,optimizers=opt,epochs=ep,batchs=bat,losses=los,metrics=met,l
oss_weights=losw,repetitions=rep)

#Execute the model MTUE with best hyperparameter combination. n iterations were used for cross-
validation

result=NULL
for(rep in 1:iterations){
  set.seed(rep)
  test=sample(1:nrow(x),nrow(x)*0.2)
  xtr = x[-test,]
  xts = x[test,]
  ytr = y[-test,]
  yts = y[test,]
  yhat=DL_MT(x,y,hyp,test=TRUE)
  result=rbind(result,diag(cor(yhat,yts)))
}
```
